# Characteristics of human papillomaviruses infection in men with genital warts in Shanghai

**DOI:** 10.18632/oncotarget.9708

**Published:** 2016-05-30

**Authors:** Xiaogang Chen, Liang Li, Yongxian Lai, Qinxiu Liu, Jianna Yan, Yichen Tang

**Affiliations:** ^1^ Department of Dermatologic Surgery, Shanghai Dermatology Hospital, Shanghai, 200442, China

**Keywords:** human papillomaviruses, male, genital warts, high-risk HPV, low-risk HPV

## Abstract

Human papillomaviruses (HPV) infected men causes continued transmission of HPV to women. The prevalence of 15 high-risk HPV strains (HPV16, 18, 31, 33, 35, 39, 45, 51, 52, 53, 56, 58, 59, 66 and 68) and 6 low-risk HPV strains (HPV6, 11, 42, 43, 44 and CP8304) were evaluated in 935 males with genital warts. Of the 447 (447/935, 47.8%) HPV DNA positive subjects, 230 (24.6%), 356 (38.1%) and 139 (14.9%) were infected by high-risk, low-risk and both high and low-risk HPV respectively. Of the 356 low-risk HPV infected subjects, 333(93.5%) were infected by single HPV strain; 203 (57.0%), 147 (41.3%), 24 (6.7%) and 5 (1.4%) were infected with HPV genotype 6, 11, CP8304 and 44 respectively; population with age ≤ 20 showed the highest infection rate. High-risk HPV are also highly prevalent in our patients, genotype 16, 58, 51, 39, 52 and 53 are the top five prevalent genotypes with infection rates of 27.4%, 18.7%, 14.3%, 13.9%, 12.6% and 12.6% respectively; only 68.3% subjects were sole infection; subjects with 41 ≤ age ≤ 50 showed the highest infection rate. Both high and low-risk HPV are highly prevalent in men with genital warts, its impact on women HPV control and prevention need further evaluation.

## INTRODUCTION

Human papillomaviruses (HPV) are small double-stranded DNA viruses that belong to the family of papillomavirus [1]. The ~8000 bp DNA genome is organized by the upstream regulatory region contains transcription regulatory elements and the origin of DNA replication; early region encodes 8 early viral proteins E1–E8; and late region encodes the major and minor capsid proteins L1 and L2 [2–4]. There are as many as 170 HPV genotypes have been identified [5]. HPV are mucosotropic and can be sexually transmitted [6]. Unique HPV genotypes were found to be disease specific, Persistent infections by the high-risk HPV genotypes leads to cancers [7]. As high as 99% of cervical cancers are believed caused by high-risk HPV, genotype 16 and 18 are responsible for about 70% of the cervical cancer cases [8]. Low-risk HPV genotypes 6 and 11 infections account for 90% of genital warts and essentially all laryngeal papillomas [9].

Genital warts are evident skin or mucosal growths in the anogenital area display as flat, papular, or pedunculated growths on the genital mucosa [10]. Genital warts occur commonly around the vaginal introitus, under the foreskin of the uncircumcised penis, and on the shaft of the circumcised penis [10]. Genital warts are one of the most common types of sexually transmitted infections highly occur in homosexual men, sex workers and human immunodeficiency virus (HIV) positive homosexual men [11]. Although genital warts are benign neoplasms, they unleash considerable harassment to patients [10]. 90% genital warts are believed to be caused by low-risk HPV genotypes 6 or 11 infection [12]. High-risk HPV genotypes 16, 18, 31, 33, and 35 are also occasionally found in genital warts and can be associated with high-grade squamous intraepithelial lesions particularly in persons who have HIV infection [12].

Treatments on genital warts are mainly divided into medications and surgery, medications include imiquimod, podophyllin, podofilox and trichloroacetic acid, surgery include freezing with liquid nitrogen, electrocautery, surgical excision and laser treatments [13, 14]. Although the short-term effect of above treatments is very good, no approach could prevent recurrence of genital warts [13]. Thus, effective prevention against HPV transmission and infection is essential. Fortunately, an effective vaccine was licensed in the year of 2006 [15]. The main components of the vaccines are virus-like particles derived from the L1 proteins of HPV 6, 11, 16, and/or 18 [15]. However, effective vaccination is largely dependent on the baseline information on HPV prevalence, genotype distribution and relative pre-existing immunity in a certain population [16].

HPV genotype distribution differs across world regions, even HPV genotypes 16, 18, 31, 52, and 58 are consistently found worldwide, the distribution of a certain HPV genotype still showed huge regional differences [17]. Therefore, to learn the HPV genotype distribution characteristics and providing matched vaccine is the prerequisite to ensure the efficiency of vaccination. On the other hands, current HPV study is mainly focused on cervical cancer relative high-risk HPV, data of HPV identified from males is relatively small. HPV infected men may act as reservoirs of HPV, resulting in continued transmission of HPV to women. In this report, patients with genital warts visited our hospital were recruited, the distribution characteristic of 15 high-risk HPV strains and 6 low-risk HPV strains in these patients were evaluated.

## RESULTS

### Summary of HPV infection

Totally, HPV DNA data of 935 male patients with genital warts were collected. 488 (488/935, 52.2%) subjects were negative for HPV DNA. 447 (447/935, 47.80%) subjects were HPV DNA positive (Table 1). Of the 447 HPV DNA positive subjects, 230 (230/935, 24.6%) and 356 (356/935, 38.1%) were infected by high-risk and low-risk HPV respectively (Table 1). 139 (139/935, 14.9%) subjects were infected by both high-risk and low-risk HPV (Table 1).

**Table 1:** Summary of HPV infection (*n* = 935)

	Number of subject	Percentage (%)
Negative	488	52.2
Total positive	447	47.8
High-risk HPV	230	24.6
High-risk HPV only	91	9.7
Low-risk HPV	356	38.1
Low-risk HPV only	217	23.2
High and low-risk HPV	139	14.9

HPV, human papillomaviruses.

### Prevalent characteristics of low-risk HPV

**Figure 1:**
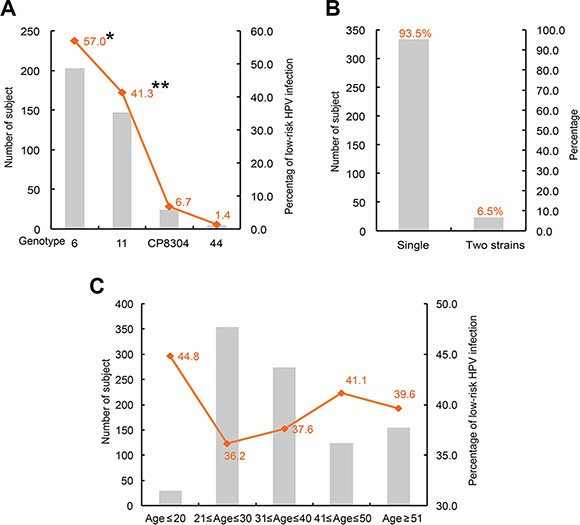
Prevalent characteristics of low-risk HPV (**A**) genotype distribution of the 356 low-risk HPV infected subjects; *P < 0.05 when compared with genotype 11, CP8304 or 44; **P < 0.05 when compared with genotype CP8304 or 44. (**B**) co-infection status of the low-risk HPV. (**C**) age distribution of low-risk HPV infection.

Of the 356 low-risk HPV infected subjects, 203 (57.0%), 147 (41.3%), 24 (6.7%) and 5 (1.4%) were infected with HPV genotype 6, 11, CP8304 and 44 respectively (Figure 1A). The frequency of genotype 11 was significantly higher than frequencies of genotype CP8304 and 44. Genotype 6 was the most dominant type, its frequency is significantly higher than the frequency of genotype 11. Of the low-risk HPV DNA detected subjects, 333(93.5%) were infected by single low-risk HPV strain, only 23 (6.5%) were infected by two low-risk HPV strains (Figure 1B). Of the age distribution, 13 (44.8%), 128 (36.2%), 103 (37.6%), 51 (41.1%) and 61(39.6%) low-risk HPV infected subjects were distributed in age ≤ 20, 21 ≤ age ≤ 30, 31 ≤ age ≤ 40, 41 ≤ age ≤ 50 and age ≥ 51 groups respectively, subjects with age ≤ 20 showed that highest infection rate (*P* < 0.05) (Figure 1C).

### Prevalent characteristics of high-risk HPV

**Figure 2:**
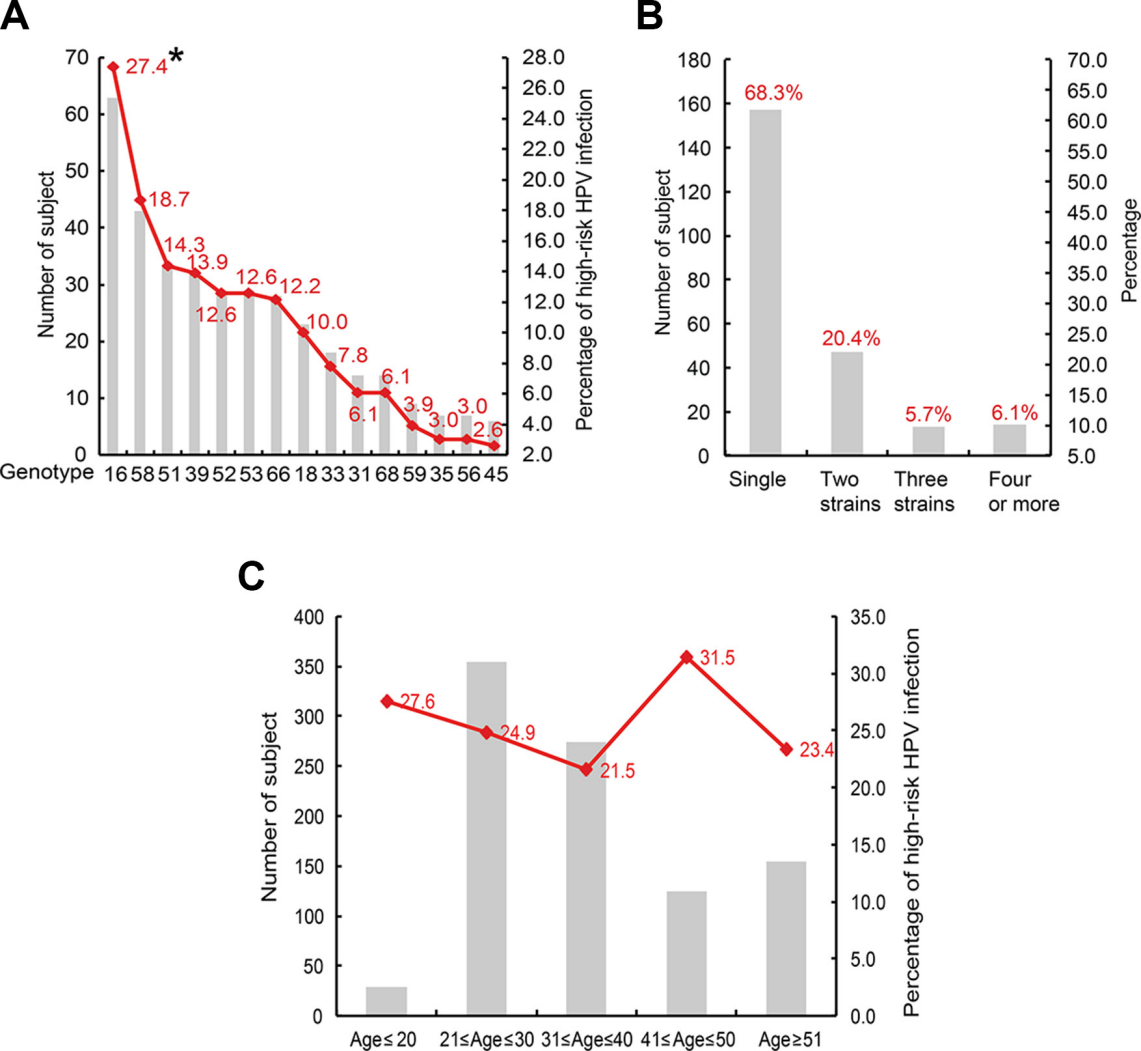
Prevalent characteristics of high-risk HPV (**A**) genotype distribution of the 230 high-risk HPV infected subjects; *P < 0.05 when compared with any other genotypes. (**B**) co-infection status of the high-risk HPV. (**C**) age distribution of high-risk HPV infection.

Of the 230 high-risk HPV infected subjects, 63 (27.4%), 43 (18.7%), 33 (14.3%), 32 (13.9%), 29 (12.6%), 29 (12.6%), 28 (12.2%), 23 (10.0%), 18 (7.8%), 14 (6.1%), 14 (6.1%), 9 (3.9%), 7 (3.0%), 7 (3.0%) and 6 (2.6%) were infected with HPV genotype 16, 58, 51, 39, 52, 53, 66, 18, 33, 31, 68, 59, 35, 56 and 45 respectively (Figure 2A). Genotype 16 was the most dominant type, its frequency is significantly higher than either other genotypes. Multiple infections with one more high-risk HPV were common, a 67 years old male was infected by as many as eight high-risk HPV strains (genotypes: 16, 33, 35, 39, 45, 51, 66 and 68). As showed in Figure 2B, 47 (20.4%), 13 (5.7%) and 14 (6.1%) subjects were infected by 2, 3, 4 or more high-risk HPV genotypes, only 157 (68.3%) subjects were sole infection. 8 (27.6%), 88 (24.9%), 59 (21.5%), 39 (31.5%) and 36 (23.4%) high-risk HPV infected subjects were distributed age ≤ 20, 21 ≤ age ≤ 30, 31 ≤ age ≤ 40, 41 ≤ age ≤ 50 and age ≥ 51 groups respectively, subjects with 41 ≤ age ≤ 50 showed the highest infection rate (*P* < 0.05) (Figure 2C).

### Co-infection HPV

To learn any preferendum of HPV co-infection, the co-infection patterns were calculated. As showed in Table 2, of the 74 multiple high-risk HPV infections, the top three co-infection genotypes for HPV16 were HPV58 (34.5%), HPV33 (31.0%) and HPV53 (20.7%); the top three co-infection genotypes for HPV58 were HPV16 (35.5%), HPV52 (26.1) and HPV53 (17.4%); and the top three co-infection genotypes for HPV51 were HPV16 (31.6%), HPV52 (21.1%) and HPV53 (21.1%). HPV16 is not only the dominant high-risk HPV in our population but also the most frequent co-infection genotype. Of the 139 subjects co-infected with both high and low-risk HPV, the rates of HPV16 co-infected with HPV11, HPV6 and CP8304 were 28.6%, 23.8% and 7.9%, respectively; the rates of HP58 co-infected with HPV6, HPV11 and CP8304 were 69.7%, 33.3% and 15.2%, respectively; and the rates of HPV51 co-infected with HPV6, HPV11 and HPV44 were 65.0%, 55.0% and 5.0%, respectively, the co-infection of high-risk HPV58 and low-risk HPV11 is the most dominant in this population with percentage as high as 69.7% (Table 2). Of the 23 multiple low-risk HPV infected subjects, the rates of HPV6 co-infected with HPV11, HPV CP8304 and HPV44 were 72.2%, 22.2% and 5.6% respectively; and the rates of HPV11 co-infected with HPV6, HPV CP8304 and HPV44 were 72.2%, 22.2% and 5.6% respectively.

**Table 2:** Characteristics of co-infections

Genotype, *N* (%)		Co-infection genotype		
*Co-infection between high-risk HPV, N = 74*				
		HPV58	HPV33	HPV53
HPV16,	29 (39.2%)	10 (34.5%)	9 (31.0%)	6 (20.7%)
		HPV16	HPV52	HPV53
HPV58,	23 (31.1%)	10 (34.5%)	6 (26.1%)	4 (17.4%)
		HPV16	HPV52	HPV53
HPV51,	19 (25.7%)	6 (31.6%)	4 (21.1%)	4 (21.1%)

*Co-infection between high and low-risk HPV, N = 139*				
		HPV11	HPV6	HPV CP8304
HPV16,	63 (27.4%)	18 (28.6%)	15 (23.8%)	5 (7.9%)
		HPV6	HPV11	HPV CP8304
HPV58	33 (23.7%)	23 (69.7%)	11 (33.3)	5 (15.2%)
		HPV6	HPV11	HPV44
HPV51	20 (14.4%)	13 (65.0%)	11 (55.0%)	1 (5.0%)

*Co-infection between low-risk HPV, N = 23*				
		HPV11	HPV CP8304	HPV44
HPV6	18 (78.3%)	13 (72.2%)	4 (22.2%)	1 (5.6%)
		HPV6	HPV CP8304	HPV44
HPV11	18 (78.3%)	13 (72.2%)	4 (22.2%)	1 (5.6%)

HPV, human papillomaviruses.

### Comparison of HPV positivity

Data regarding HPV infection in men is relatively rare and not comprehensive, to compare HPV positivity in this and other studies, we summarized the representative published data. The data include three cross-country studies and one from Brazil [18–21]. As showed in the Table 3, HPV infection profile in men with genital warts varied among reports, even within the three cross-country studies, their data also varied greatly. HPV infection is highly prevalent in Brazil. HPV genotype distribution displayed regional difference.

**Table 3:** Comparison of HPV positivity in this and other studies

Genotype	Our data	Cross-country study [18]	Cross-country study [19]	Cross-country study [20]	Brazil [21]
*High-risk HPV*					
16	27.4	4.5	3.8	9.8	11.8
58	18.7	0.5	1.7	3.6	3.9
51	14.3	5.6	3.9	5.4	11.0
39	13.9	1.9	2.2	4.5	8.7
52	12.6	5.6	3.1	6.2	5.9
53	12.6	1.3	/	7.1	8.7
66	12.2	1.3	/	5.4	6.5
18	10.0	0.8	2.0	3.6	2.8
33	7.8	0.8	0.7	0.0	4.5
31	6.1	2.1	1.7	0.9	5.6
68	6.1	0.5	/	2.7	6.8
59	3.9	0.0	2.5	4.5	5.9
35	3.0	0.0	1.0	0.0	1.1
56	3.0	0.3	3.6	0.9	7.6
45	2.6	0.3	1.4	0.9	1.4
*Any tested type*	*24.6*	*15.6*	*/*	*/*	*47.4*
*Low-risk HPV*					
6	57.0	47.5	3.4	43.8	17.7
11	41.3	25.2	5.0	10.7	5.9
CP8304	6.7	/	/	/	/
44	1.4	2.1	/	/	9.0
*Any tested type*	*38.1*	*73.2*	*/*	*/*	*52.6*

The data was presented as percentage. /data not avaliable.

## DISCUSSION

HPV, as one of the seven notorious carcinogenic viruses, is being studied worldwide [22]. Recently, significant progress in the control and prevention on HPV infection had made, the success of prophylactic HPV vaccines is the best case in point [23]. However, the genotypes of HPV are at least as many as 170 and the distribution of these genotypes differed regionally [5, 17], vaccines can not ensure to protect worldwide people sufficiently regardless of octavalent or bivalent vaccines. Thus, surveillance on HPV genotype distribution possesses the same importance as vaccine development, regimen modification and dosage improvement. Low-risk HPV could cause genital warts and laryngeal papillomas, so, the study on low-risk HPV is easy to be overlooked. In fact, burden of low-risk HPV is also huge, HPV is the most common genital infection in the US, and the lifetime risk of at least one HPV infection in women is 75% [24, 25]. Further more, HPV infected men may act as reservoirs of HPV, resulting in continued transmission of HPV to women. Thus, surveillance on HPV infection in males is also important.

In this study, we focused on the HPV prevalence of 15 high-risk HPV strains, HPV16, 18, 31, 33, 35, 39, 45, 51, 52, 53, 56, 58, 59, 66 and 68 and 6 low-risk HPV strains, HPV6, 11, 42, 43, 44 and CP8304 in males with genital warts. Our data showed that, 47.8% subjects were HPV DNA positive, 24.6%, 38.1% and 14.9% of the HPV DNA positive subjects were infected by high-risk, low-risk and high plus low-risk HPV, the prevalence of high-risk HPV is also high in population with genital warts. Secondly, of low-risk HPV infection, HPV 6 and 11 were the dominant genotypes in our population, they account for 93.3% infection; multiple infection among low-risk HPV is relative low, 93.5% were infected by single strain; age ≤ 20 subjects showed that highest infection rate (44.8%). Thirdly, of high-risk HPV infection, HPV 16, 58, 51, 39 and 52 are the top five dominant genotypes, multiple infections with high-risk HPV are common, only 68.3% subjects were sole infection; subjects with 41 ≤ age ≤ 50 showed the highest infection rate (31.5%). Finally, HPV16 is not only the dominant high-risk HPV in our population but also the most frequent co-infection genotype.

Although current opinion believe that the 90% genital warts are caused by two low-risk HPV subtypes 6 and 11 [9], the real situation regarding the prevalence and genotype distribution of low-risk HPV in population with genital warts is rarely reported. A recent systematic review of genital HPV among men in sub-Saharan Africa showed that the prevalence of any HPV genotype ranges between 19.1% and 100% [26, 27]. Our data showed that the over all HPV infection rate is 47.8%. Although it is believed that sexually activity, homosexual and heterosexual might impact the HPV prevalence [26], a recent global review on age-specific prevalence of HPV infection in males had not supported this opinion [28]. Our data showed that although HPV 6 and 11 account for as high as 93.3% proportion of the low-risk HPV infected subjects, the low-risk HPV DNA was detected only in 38.1% of our patients. Actually, HPV infection profile in men with genital warts varied among reports, even within the three cross-country studies, their data also varied greatly [18–20]. We ensure the operation specification in genital warts sampling and the quality control of HPV DNA detection, so this difference might be explained by the following reasons: firstly, the natural history of HPV infection, individual might be infected by multiple low-risk HPV strains in his life, single time point sampling could not capture the whole history of HPV infection [29]; secondly, the 6 generally accepted highly prevalent low-risk HPV might not represent the real situation of low-risk HPV infection, many other HPV genotypes might be prevalent highly; thirdly, the real impact of low-risk HPV infection on genital warts is not that high as we known.

The prevalence of both low-risk HPV and high-risk HPV displayed differences in age groups, this phenomenon was also observed in the HPV infection in women [29, 30]. Although we are not sure what cause these differences, our data showed that HPV infection in men with genital warts was throughout the life span.

Based on our data, the high-risk HPV are also highly prevalent in the population with genital warts, there are two important information of high-risk HPV infection in our male patients, firstly, since HPV infection is a sexually transmitted disease, efficient HPV control and prevention in women could not ignore that high HPV infections in male; secondly, our data showed that HPV 16, 58, 51, 39 and 52 are the top five dominant genotypes, which differed from the data from a meta-analysis with a large-scale women population, in that report, HPV 16, 18, 31, 52, and 58 are consistently found among the 10 most common types in 5 continents [17], this difference might be explained by gender difference, but on the other hand, we could not eliminate the real difference exists between our population and populations pooled in that meta-analysis. In conclusion, any impact of high-risk HPV on the pathophysiological process of genital warts should be valued in further clinical trails.

## MATERIALS AND METHODS

### Ethic issues

This study was conducted in accordance with the World Medical Association Declaration of Helsinki (http://www.who.int/bulletin/archives/79(4)373.pdf). The Review Board of the Ethics Committee of Medical Research at Shanghai Dermatology Hospital (1278 Baode Road, Jingan District, Shanghai) approved the study protocols. Written informed consents were obtained from all patients according to the guidelines of the Chinese National Ethics Regulation Committee. All patients were informed of their rights to withdraw consent personally or via kin, caretakers, or guardians.

### Subjects

To learn the characteristics of HPV infection in males with genital warts of Shanghai, a cross-sectional study was adopted. Adult male patients with genital warts were recruited sequentially from January 2014 to May 2015 at our Hospital. Diagnosis of genital warts is made by visual inspection. When lesions are atypical, e.g., pigmented, indurated, affixed to underlying tissue, bleeding, or ulcerated lesions, the diagnosis was confirmed by biopsy. The exclusion criteria include patients with uncertain diagnosis and patients undergoing standard therapy. Finally, 935 out of 1114 patients were included in this study, the median age and quartile range were 42 and 31 to 56.

### HPV testing

Biopsies of the outer genital area (scrotum or penis) were performed under local anesthetic. Biopsy samples or surgical specimens were subjected to clinical laboratory for pathological examination. Simultaneously, a part of the biopsy sample of each patient was used for HPV DNA extraction after grinding. DNA extractions were performed using QIAmp Mini Kit (Qiagen, Shanghai, China), a water blank was processed through all steps of extraction to serve as a contamination control. The extract was stored at –20^O^C before HPV detection. HPV DNA testing and HPV genotyping was performed using commercial detection kit purchased from Hybribio (Hongkong, China). The kit uses polymerase chain reaction (PCR) with biotinylated PGMY09/11 primer sets that target HPV L1 consensus region, beta-globin was adopted as an internal control for sample amplification. All PCR products were hybridized to the typing strip that included probes for 15 high-risk HPV strains (HPV16, 18, 31, 33, 35, 39, 45, 51, 52, 53, 56, 58, 59, 66 and 68) and 6 low-risk HPV strains (HPV6, 11, 42, 43, 44 and CP8304). All detection procedures were guided by the manufacturers protocol. Detected HPV strains are defined by genotype not by sub-genotype or quasispecies. The definitions of high-risk HPV and low-risk HPV genotypes were basically based on the Human Papillomavirus Vaccination, Recommendations of the Advisory Committee on Immunization Practices (ACIP) [31]. According to the etiology of cervical cancer, high-risk HPV genotypes have the potential to act as carcinogens; low-risk HPV genotypes can cause benign or low-grade cervical cell changes, genital warts, and recurrent respiratory papillomatosis [31].

### Statistical analysis

Statistical analysis was performed using SPSS version 13.0. Continuous variables were presented as means ± standard deviation (SD) and categorical data were presented as numbers (percentage). Differences between groups were examined by using *t*-tests, one-way ANOVA, χ^2^ or Fisher Exact Probability Test according to the characteristics of data distribution. The significance level α was set at 0.05.

## References

[1] Woodman CB, Collins SI, Young LS (2007). The natural history of cervical HPV infection: unresolved issues. Nat Rev Cancer.

[2] Chow LT, Broker TR (2013). Human papillomavirus infections: warts or cancer. Cold Spring Harb Perspect Biol.

[3] Bian T, Wang Y, Lu Z, Ye Z, Zhao L, Ren J, Zhang H, Ruan L, Tian H (2008). Human papillomavirus type 16 L1E7 chimeric capsomeres have prophylactic and therapeutic efficacy against papillomavirus in mice. Mol Cancer Ther.

[4] Chow LT, Broker TR, Steinberg BM (2010). The natural history of human papillomavirus infections of the mucosal epithelia. APMIS.

[5] Ghittoni R, Accardi R, Chiocca S, Tommasino M (2015). Role of human papillomaviruses in carcinogenesis. Ecancermedicalscience.

[6] Bravo IG, de Sanjosé S, Gottschling M (2010). The clinical importance of understanding the evolution of papillomaviruses. Trends Microbiol.

[7] zur Hausen H (2009). Papillomaviruses in the causation of human cancers - a brief historical account. Virology.

[8] Walboomers JM, Jacobs MV, Manos MM, Bosch FX, Kummer JA, Shah KV, Snijders PJ, Peto J, Meijer CJ, Muñoz N (1999). Human papillomavirus is a necessary cause of invasive cervical cancer worldwide. J Pathol.

[9] Derkay CS1, Wiatrak B (2008). Recurrent respiratory papillomatosis: a review. Laryngoscope.

[10] Steben M, Garland SM (2014). Genital warts. Best Pract Res Clin Obstet Gynaecol.

[11] Maw R (2006). Anogenital warts. Sex Transm Infect.

[12] Garland SM, Steben M, Sings HL, James M, Lu S, Railkar R, Barr E, Haupt RM, Joura EA (2009). Natural history of genital warts: analysis of the placebo arm of 2 randomized phase III trials of a quadrivalent human papillomavirus (types 6, 11, 16, and 18) vaccine. J Infect Dis.

[13] Leszczyszyn J, Łebski I, Łysenko L, Hirnle L, Gerber H (2014). Anal warts (condylomata acuminata) - current issues and treatment modalities. Adv Clin Exp Med.

[14] Fathi R, Tsoukas MM (2014). Genital warts and other HPV infections: established and novel therapies. Clin Dermatol.

[15] Stokley S, Jeyarajah J, Yankey D, Cano M, Gee J, Roark J, Curtis RC, Markowitz L (2014). Immunization Services Division National Center for Immunization Respiratory Diseases CDC; Centers for Disease Control and Prevention (CDC) Human papillomavirus vaccination coverage among adolescents, 2007–2013, and postlicensure vaccine safety monitoring, 2006–2014—. MMWR Morb Mortal Wkly Rep.

[16] Wang W, Peng H, Zhao P, Qi Z, Zhao X, Wang Y, Wang C, Hang X, Ke J (2014). Cross-reactive antibody responses to the novel avian influenza A H7N9 virus in Shanghai adults. J Infect.

[17] Bruni L, Diaz M, Castellsagué X, Ferrer E, Bosch FX, de Sanjosé S (2010). Cervical human papillomavirus prevalence in 5 continents: meta-analysis of 1 million women with normal cytological findings. J Infect Dis.

[18] Ingles DJ, Pierce Campbell CM, Messina JA, Stoler MH, Lin HY, Fulp WJ, Abrahamsen M, Sirak BA, O'Keefe MT, Papenfuss M, Gage C, Carvalho da Silva R, Gonzalez Sosa R (2015). Human papillomavirus virus (HPV) genotype- and age-specific analyses of external genital lesions among men in the HPV Infection in Men (HIM) Study. J Infect Dis.

[19] Vardas E, Giuliano AR, Goldstone S, Palefsky JM, Moreira ED, Penny ME, Aranda C, Jessen H, Moi H, Ferris DG, Liaw KL, Marshall JB, Vuocolo S (2011). External genital human papillomavirus prevalence and associated factors among heterosexual men on 5 continents. J Infect Dis.

[20] Anic GM, Lee JH, Stockwell H, Rollison DE, Wu Y, Papenfuss MR, Villa LL, Lazcano-Ponce E, Gage C, Silva RJ, Baggio ML, Quiterio M, Salmerón J (2011). Incidence and human papillomavirus (HPV) type distribution of genital warts in a multinational cohort of men: the HPV in men study. J Infect Dis.

[21] Freire MP, Pires D, Forjaz R, Sato S, Cotrim I, Stiepcich M, Scarpellini B, Truzzi JC (2014). Genital prevalence of HPV types and co-infection in men. Int Braz J Urol.

[22] Moore PS, Chang Y (2010). Why do viruses cause cancer? Highlights of the first century of human tumour virology. Nat Rev Cancer.

[23] Schiller JT, Lowy DR (2012). Understanding and learning from the success of prophylactic human papillomavirus vaccines. Nat Rev Microbiol.

[24] Baseman JG, Koutsky LA (2005). The epidemiology of human papillomavirus infections. J Clin Virol.

[25] Cates W (1999). Estimates of the incidence and prevalence of sexually transmitted diseases in the United States. American Social Health Association Panel. Sex Transm Dis.

[26] Olesen TB, Munk C, Christensen J, Andersen KK, Kjaer SK (2014). Human papillomavirus prevalence among men in sub-Saharan Africa: a systematic review and meta-analysis. Sex Transm Infect.

[27] Banura C, Mirembe FM, Orem J, Mbonye AK, Kasasa S, Mbidde EK (2013). Prevalence, incidence and risk factors for anogenital warts in Sub Saharan Africa: a systematic review and meta analysis. Infect Agent Cancer.

[28] Smith JS, Gilbert PA, Melendy A, Rana RK, Pimenta JM (2011). Age-specific prevalence of human papillomavirus infection in males: a global review. J Adolesc Health.

[29] Gu Y, Yi M, Xu Y, Zhao H, Fu F, Zhang Y (2016). Genotype distribution characteristics of high-risk human papillomaviruses in women from Shanghai, China. Epidemiol Infect.

[30] Gu Y, Ma C, Zou J, Zhu Y, Yang R, Xu Y, Zhang Y (2016). Prevalence characteristics of high-risk human papillomaviruses in women living in Shanghai with cervical precancerous lesions and cancer. Oncotarget.

[31] Markowitz LE, Dunne EF, Saraiya M, Chesson HW, Curtis CR, Gee J, Bocchini JA (2014). Unger ER; Centers for Disease Control and Prevention (CDC). Human papillomavirus vaccination: recommendations of the Advisory Committee on Immunization Practices (ACIP). MMWR Recomm Rep.

